# Genetic diversity in Kashubs: the regional increase in the frequency of several disease-causing variants

**DOI:** 10.1007/s13353-022-00713-z

**Published:** 2022-08-15

**Authors:** Maciej Jankowski, Patrycja Daca-Roszak, Cezary Obracht-Prondzyński, Rafał Płoski, Beata S. Lipska-Ziętkiewicz, Ewa Ziętkiewicz

**Affiliations:** 1 Department of Biology and Medical Genetics, Medical University of Gdansk, Gdansk, Poland; 2 Institute of Human Genetics, Polish Academy of Sciences, Poznan, Poland; 3 Institute of Sociology, University of Gdansk, Gdansk, Poland; 4 Department of Medical Genetics, Medical University of Warsaw, Warsaw, Poland; 5 Clinical Genetics Unit, Department of Biology and Medical Genetics, Medical University of Gdansk, Gdansk, Poland; 6 Centre for Rare Diseases, Medical University of Gdansk, Gdansk, Poland

**Keywords:** Founder mutation, Rare diseases, Polish population structure, Demography, Kashubian

## Abstract

Differential distribution of genetic variants’ frequency among human populations is caused by the genetic drift in isolated populations, historical migrations, and demography. Some of these variants are identical by descent and represent founder mutations, which — if pathogenic in nature — lead to the increased frequency of otherwise rare diseases. The detection of the increased regional prevalence of pathogenic variants may shed light on the historical processes that affected studied populations and can help to develop effective screening and diagnostic strategies as a part of personalized medicine. Here, we discuss the specific genetic diversity in Kashubs, the minority group living in northern Poland, reflected in the biased distribution of some of the repetitively found disease-causing variants. These include the following: (1) c.662A > G (p.Asp221Gly) in *LDLR*, causing heterozygous familial hypercholesterolemia; (2) c.3700_3704del in *BRCA1*, associated with hereditary breast and ovarian cancer syndrome; (3) c.1528G > C (p.Glu510Gln) in *HADHA*, seen in long-chain 3-hydroxy acyl-CoA dehydrogenase (LCHAD) deficiency, and (4) c.1032delT in *NPHS2*, associated with steroid-resistant nephrotic syndrome.

## Introduction

The genetic diversity of human populations is shaped by the occurrence of mutations, selection, and genetic drift [Przeworski et al. 2000]. The genetic drift — random changes in allele frequencies in isolated populations — depends on populations’ demography and migrations [Veeramah & Novembre 2014]. As a consequence, the historical processes that affected studied populations may result in specific regional patterns of the genetic diversity, both neutral and manifesting as genetic diseases. Inter-population differences in the frequency of variants underlying rare genetic diseases are of special interest because of their relevance for diagnostic strategies.

Some of the deleterious variants observed at the increased frequency in certain populations represent the so-called founder mutations [e.g., Prohaska et al. 2019]. Contemporary alleles carrying a founder variant share a common ancestor and are identical by descent; an alternative scenario, when the increased frequency of a genetic variant results from independent recurrent mutations at the genomic hot-spot, is not considered here. To prove that the present-day chromosomes indeed share a common origin, the haplotype background of a founder mutation can be analyzed; this also allows determining the approximate time of the variant’s introduction into the studied population. Different methods relying on the recombinational decay of the ancestral haplotype [e.g., Labuda et al. 1996; Gandolfo et al. 2014], can be used to infer its age, but their description goes beyond the scope of this review. When combined with historical and genetic data from the relevant human groups, haplotype analysis may indicate the time and the population, in which the founder mutation originated [Labuda et al. 1997; Yotova et al. 2005; Greenwood et al. 2010; Hamel et al. 2011].

## The genetic diversity of the Polish population

Until World War II (WWII), Poland was characterized by a strong ethnic diversification; in 1931, Poles constituted about 69% of the Polish population. War operations as well as forced relocations after WWII significantly changed the ethnic structure of Poland. The previous mosaic structure has disappeared, replaced by the nationally and ethnically homogeneous population [Maryanski 1998]. According to the 2011 census, Poles constitute about 97.1% of 38.5 mln inhabitants [Gudaszewski GUS 2011]. In 2005, Poland adopted the Act on National and Ethnic Minorities and Regional Language [ACT 2005], according to which there are four recognized ethnic minorities (Lemkos, Roma, Karaims, and Tatars), and nine national minorities, the most numerous being German, Ukrainian, Jewish, and Russian. According to the 2011 census, slightly more than 870,000 people belong to the state-recognized minorities, the most numerous being German and Ukrainian. While most of the self-recognized minority declarations concern Silesians, they are not a minority recognized by the Polish state. On the other hand, Kashubs are not an ethnic or national minority, but they are the only legally recognized community that uses the regional language [ACT 2005].

This low ethnic diversity in the present-day population of Poland is reflected by its genetic homogeneity. It is supported by numerous studies: on blood group systems [Gronkiewicz 1996], human leukocyte antigen (HLA) haplotypes [Schmidt et al. 2013], the Y-chromosome lineages [Płoski et al. 2002; Rębała et al. 2013], major mtDNA haplogroups [Malyarchuk et al. 2002; Grzybowski et al. 2007; Mielnik-Sikorska et al. 2013; Jarczak et al. 2019], the forensic short tandem repeat (STR) markers [Sołtyszewski et al. 2008], and recently by whole-genome sequencing (WGS) data [Kaja et al. 2021].

Even in nationally homogeneous populations, like Poland, some degree of ethnic, historical, or cultural isolation exists. This is often paralleled by the specific genetic pool composition that distinguishes local human groups from the rest of the people inhabiting a given territory. Many studies have demonstrated regional differences in the occurrence of some genetic variants within the Polish population, with various scenarios proposed to explain them [Witt et al. 1996; Jagodziński et al. 2000; Grzybowski et al. 2007]. Some differences in the distribution of rare deleterious variants across Poland have been described to represent regional founder mutations. Unfortunately, many of the studies on the rare variants’ distribution did not include samples from Polish minorities; therefore, some of the local differences could have been missed. Kashubs provide an example of a minority population, in which a local increase of the frequency of several pathogenic variants has been revealed only thanks to including this ethnically distinct group in population screening efforts.

## Kashubs — an ancient ethnic group embedded in the homogeneous Polish population

**Fig. 1:**
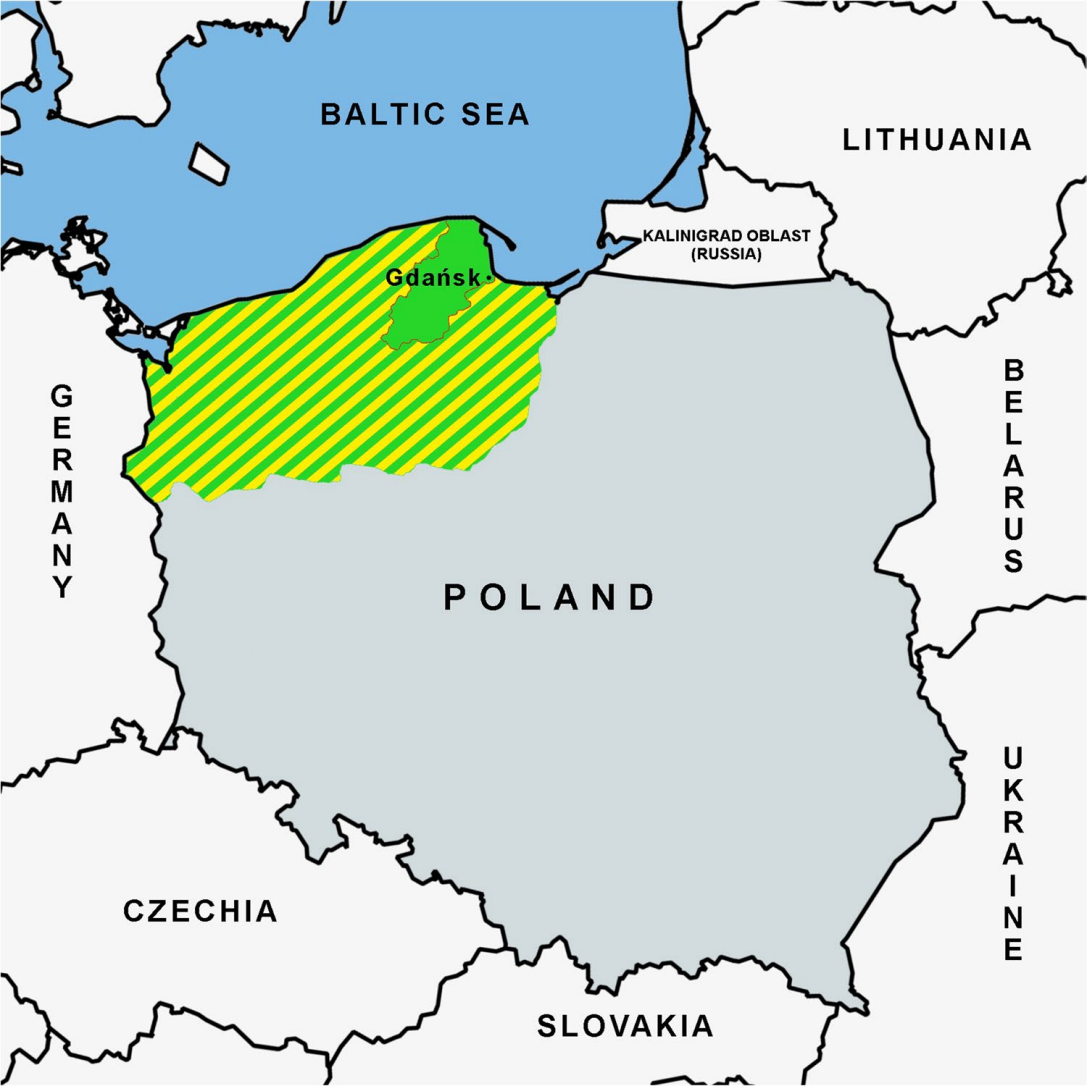
The region of Kashubia. Legend: Green color: communities where Kashubs are presently the dominant (30–90%) ethnic group; shaded green/yellow: maximal extension of the historical Pomerania/Kashubia on the South of Baltic sea in the Northern Poland

Kashubs are the West-Slavic ethnic group living today in northern Poland, in the region of Pomerania (Fig. 1). Their ancestors came to this area, earlier inhabited by German tribes, during the early medieval period of a demographic expansion of the Slavic people [Mordawski 2017]. Pomeranian language groups (since the thirteenth century referred to as Kashubs) inhabited the entire Pomerania between the Odra (Oder) and Wisła (Vistula) rivers. They never created one state, and their fates were different in the western and eastern parts of the region. For centuries, Pomerania was at the cultural borderland, under the competing German and Slavic influence. Between the fourteenth and sixteenth century, the German influence prevailed in the western part. In the sixteenth century, Kashubs inhabiting this region became Protestants and by the beginning of the twentieth century were almost completely assimilated into German culture. In the eastern part of Pomerania (which was part of Poland from the fifteenth century until the partitions of 1772), Kashubs retained their separate identity, language, and culture [Labuda 2006; Obracht-Prondzyński & Wicherkiewicz 2012; Mordawski 2017]. At the end of the twentieth century, the number of Kashubs living in eastern Pomerania was 350,000–380,000 [Mordawski 1999]. In the 2011 census, over 232,000 Polish citizens declared Kashubian identification (over 215,000 both Polish and Kashubian, and over 16,000 only Kashubian); in addition, over 108,000 confirmed using the Kashubian language in their home contacts. The nineteenth–twentieth century migrations from eastern to western European territories and to the Americas involved Kashubs as well, but this did not change the identity of the Kashubian population who remained settled in eastern Pomerania [Obracht-Pradzynski, 2020].

Studies on the neutral genetic variation have shown that the contemporary genetic profile of Kashubs in eastern Pomerania differs from that in the neighboring regions. The analysis of the mitochondrial DNA diversity in Ukrainians, Czechs, and Poles has indicated that three haplogroups (J1, T*, and H10) are more frequent in Kashubs than in Podhalans from southern Poland, or in populations from two neighboring countries [Mielnik-Sikorska et al. 2013]. The study of the Y chromosome lineages carried out in modern Poles has failed to reveal genetic differentiation among different Polish groups, including Kashubs [Woźniak et al. 2010]. However, another study of the Y-chromosome diversity in populations from northern Poland, Germany, and Czechs/Slovakia has shown that the paternal lineages in the pre-WWII Poland were unevenly distributed, so the observed present-day homogeneity may be the result of the massive post-war resettlements [Rębała et al. 2013].

The genetic distinctiveness of Kashubs can be seen in the prevalence of some rare genetic diseases: familial hypercholesterolemia, heritary breast and ovarian cancer syndrome, long-chain 3-hydroxyacyl-CoA dehydrogenase deficiency, and steroid-resistant nephrotic syndrome. Several studies have reported that certain pathogenic variants occur at an increased frequency in the region inhabited by Kashubs compared to other parts of Poland and Europe (Table 1; discussed in detail below).

**Table 1:** The general population frequency of the disease-causing variants frequently reported in Kashubian patients

Disease	Inheritance	Gene	Variant	Allele freq. worldwide^a^	Europe^a^	North-western Europe^a^	South Europe^a^	Poland^b^
Familal hypercholesterolemia (FH)	dominant	*LDLR* (chr.19)	NM_000527.5:c.662A > G (p.Asp221Gly)	0.000052 13/249,796	0.000115 13/112,856	0,000,048 2/41,868	0.000699 8/11,446	0
Hereditary breast and ovarian cancer syndrome (HBOCS)	dominant	*BRCA1* (chr.17)	NM_007294.4:c.3700_3704del (p.Val1234GlnfsTer8)	0	0	0	0	0
Long-chain 3-hydroxyacyl-CoA dehydrogenase deficiency (LCHADD)	recessive	*HADHA* (chr. 2)	NM_000182.5:c.1528G > C (p.Glu510Gln)	0.0012298 309/251,462	0.001521 173/113,738	0.001280 54/42,204	0.000609 7/11,496	0.001949 9/4618
Steroid-Resistant Nephrotic syndrome (SRNS)	recessive	*NPHS2* (chr. 1)	NM_014625.4:c.1032del (p.Phe344LeufsTer4)	0	0	0	0	0

^a^After GNOMAD v2.1.1 exome database (https://gnomad.broadinstitute.org/ accessed February 2022); a data set comprising exome sequences from 125,748 unrelated individuals, sequenced as part of various disease-specific and population genetic studies worldwide. Data for Europe without population from Finland

^b^In-house exome database of 2309 consecutive Polish individuals (4618 chromosomes) sequenced in the Department of Medical Genetics, Medical University of Warsaw, Poland

## Familial hypercholesterolemia (FH); *LDLR* c.662A > G.

Familiar hypercholesterolemia (FH, ORPHA: 406) is an autosomal dominant disorder, which in Europe occurs at the frequency of ~ 1:250–1:700. In ~ 85% of cases, FH is caused by heterozygous pathogenic variants in the low-density lipoprotein receptor gene, *LDLR* (OMIM #143890); less often, by heterozygous variants in the apolipoprotein B-100 gene, *APOB* (OMIM #144010) or in the proprotein convertase subtilisin/kexin type 9 gene, *PCSK9* (OMIM #603776) [Youngblom et al. 1993]. Also, rare cases of biallelic familial hypercholesterolemia are known, but they occur at a much lower frequency (< 1:100,000; ORPHA: 391,665).

The spectrum of *LDLR* disease-causing variants in Europe varies between countries, from a few to hundreds of distinct mutations responsible for FH; the frequencies of some of these variants in certain populations are elevated due to founder effects [Dedoussis et al. 2004]. In several genetic screens performed in the Polish population, a single *APOB* variant and tens of pathogenic variants in *LDLR* have been reported [Górski et al. 1998; Plewa et al. 2006; Chmara et al. 2010; Mickiewicz et al. 2016; Sharifi et al. 2016]. The *LDLR* variants most frequently found in Polish FH patients include the following: NM_000527.5:c.1775G > A in exon 12 (p.Gly592Glu),^1^ c.662A > G in exon 4 (p.Asp221Gly), and two large rearrangements, duplication of exons 4–8, and deletion of exons 5–10. The reported proportion of patients carrying these variants varies across these studies, due to different criteria of cohort composition and because of using different denominators (the whole FH cohort versus a subgroup with *LDLR/APOB* mutations). Here, we present the comparison of two cohorts composed of consecutively collected FH patients in two studies, from northern to south-eastern Poland [Mickiewicz et al. 2016; Sharifi et al. 2016] (Table 2).

**Table 2:** The most frequent disease-causing variants among Polish FH patients

Regionof Poland	Northern [Mickiewicz et al. 2016]	South-eastern [Sharifi et al. 2016]
FH cohort size	193	161
# patients with *LDLR* or *APOB* disease-causing variants % of FH cohort	80 41.5	70 43.5
*LDLR* c.1775G > A; p.Gly592Glu		
# patients	6	4
% of *LDLR/APOB* subgroup	7.5	5.7
% of the whole FH cohort	3.1	2.5
*LDLR* c.662A > G; p.Asp221Gly		
# patients*	13	0
% of *LDLR/APOB* subgroup	16.3	-
% of the whole FH cohort	6.7	-
*LDLR* dup ex 4–8		
# patients	4	2
% of *LDLR/APOB* subgroup	5.0	2.9
% of the whole FH cohort	2.1	1.2
*LDLR* del ex 5–10		
# patients	0	6
% of *LDLR/APOB* subgroup	-	8.6
% of the whole FH cohort	-	3.7
*APOB* c.10580G > A; p.Arg3527Gln		
# patients	12	13
% of *LDLR/APOB* subgroup	15.0	18.5
% of the whole FH cohort	6.2	8.1

*11/13 individuals from Pomerania harboring this variant were Kashubs.

The c.662A > G variant has been found in 13 out of 193 FH patients from Northern Poland, the majority of them (11/13) are of Kashubian origin [Mickiewicz et al. 2016], preliminarily reported in Chmara et al. (2010). No individuals with c.662A > G have been found in south-eastern Poland [Sharifi et al. 2016]. The frequency of Polish FH individuals harboring c.662A > G is therefore 6.7%, but only for the northern part of the country. The c.662A > G variant has been previously reported in patients from other — but not all — European populations [Dedoussis et al. 2004]. In the study of 725 unrelated FH patients from Italy, 6.6% of individuals have been found to harbor this variant [Bertolini et al. 2000]. Interestingly, c.662A > G formed clusters of the elevated frequency in the northern (36% of FH families) but not central or southern parts of the country; the shared haplotype confirmed the common origin of this variant in northern Italy [Bertolini et al. 2000]. The c.662A > G variant has also been reported in Austria (20 individuals among 950 unrelated FH patients; frequency 2.1%) [Schmidt and Kostner 2000], and Croatia (a single case among 420 FH patients; 0.2%) [Rukavina et al. 2001].

The c.662A > G variant observed in Pomerania could have arisen as a new independent mutation in a Kashubian ancestor or been imported by an individual from a distant population (presumably, Italian or Austrian). Determination of the underlying haplotype would be necessary to estimate the time and the plausible historical scenario of the c.662A > G introduction into the region. In any case, with the elevated frequency in eastern Pomerania and the absence in other regions of Poland, c.662A > G can be considered a founder mutation in Kashubs. Its local propagation in the descendants of the founder could be related to the survival advantage conferred by hypercholesterolemia in face of infectious diseases [Sijbrands et al. 2001].

## Hereditary breast and/or ovarian cancer syndrome (HBOCS); *BRCA1* c.3700_3704del

Approximately 5–10% of breast cancers are attributable to genetic susceptibility. Hereditary breast and ovarian cancer syndrome (HBOCS, ORPHA: 145) is associated with pathogenic variants in several predisposing genes, which are transmitted in an autosomal dominant manner. To date, over 3500 pathogenic sequence variants in two major HBOCS susceptibility genes, *BRCA1* (OMIM #604370) and *BRCA2* (OMIM #612555) have been described [Rebbeck et al. 2018].

The overall frequency of individuals harboring deleterious *BRCA1* variants has been estimated at 0.1–0.3% in most European and American countries. However, in some populations, the frequency of few variants is significantly increased, reflecting the presence of regional founder mutations [Rebbeck et al. 2018].⁠

In Poland, there are several repetitively found pathogenic variants in *BRCA1*^2^ (Table 3). The reported proportion of patients carrying *BRCA1* variants varies across the studies, due to different criteria of cohort composition (hereditary breast cancer, hereditary ovarian cancer, hereditary breast and/or ovarian cancer) or different stringency of inclusion criteria (consecutive patients, severe cases, age of onset, etc.). Therefore, while discussing variant frequencies, it is essential to indicate whether it is based on the analysis of a group of patients or on the general population.

**Table 3:** Frequency of the disease-causing *BRCA1* variants most frequent in Polish population (see text for the references)

	Whole country	Pomerania	European occurrence	Founder effect
c.5266dupC			Pan-European	in Ashkenazim
% HBOCS patients	1.9–2.1			
% in general population	0.17–0.20	0		
c.181 T > G (p.Cys61Gly)			Central-European	in Ashkenazim
% HBOCS patients	0.7–1.0			
% in general population	0.05–0.08	0.12		
c.68_69del			Rare in non-Jews	in Ashkenazim
# HBOCS patients	0.5–5.7			
# in general population	Not reported			
c.4035del			Baltic countries	
% HBOCS patients	< 1			
% in general population	Not reported			
c.3700_3704del			Various populations	in Czechs
% patients	1.7–6.3			
% in general population	0.1*	1.17		

*Reported only in Pomerania.

The two most frequent pathogenic variants, NM_007294.4:c.5266dupC and c.181 T > G (p.Cys61Gly), collectively account for 60–80% of *BRCA1* disease-causing variants detected in Polish patients [Brożek et al. 2011]. The c.5266dupC is a pan-European variant; haplotype studies have shown that it most likely originated ~ 1800 years ago in Scandinavia or Northern Russia and spread throughout Europe; a subsequent founder effect in Ashkenazi Jews, dated at ~ 400–500 years ago, is responsible for its elevated frequency in this population [Struewing et al. 1997; Hamel et al. 2011]. In Poland, c.5266dupC has been reported in 2.1% [Górski 2006] or 1.9% of consecutive breast cancer patients [Brożek et al. 2011], and in 0.20% or 0.17% of the general Polish population [Górski 2006; Brożek et al. 2011]; it is more frequent in the north-eastern region of Warmia-Mazury (0.42%) than in Pomerania (0%) or other Polish provinces (0.13%) [Brożek et al. 2011]. ⁠

The c.181 T > G variant is prevalent and considered a founder mutation throughout Central Europe [Csokay et al. 1999; Meindl 2002; Kaufman et al. 2009; Elsakov et al. 2010]. In Poland, the frequency of individuals harboring c.181 T > G has been estimated at 0.7% [Górski 2006] or 1.0% [Brożek et al. 2011] among consecutive breast cancer patients, and at 0.05% or 0.08% in the general population, with no significant differences across the provinces.

Other *BRCA1* variants repetitively found in the Polish population include c.68_69del, c.4035del, and c.3700_3704del. None of the first two variants has been found in the large-scale studies of the general Polish population [Górski 2006; Brożek et al. 2011]. The frequency of c.68_69del (the Ashkenazi founder variant) among Polish HBOCS families has been reported to range from 0 to 5.7%, depending on the variably defined study groups [Hartwig et al. 2013 and references therein; Kowalik et al. 2018]. The c.4035del variant, which presumably originated in Lithuania and spread in the Baltic Sea countries [Janavicius et al. 2012], has been found among Polish breast cancer patients with a frequency of less than 1% [Górski 2006; Brożek et al. 2008; Kowalik et al. 2018].

The c.3700_3704del is a founder mutation in Czechs (reported in ~ 3% of breast cancer patients) [Machackova et al. 2008]; it is also found in other European populations, including Greek [Konstantinopoulou et al. 2014], Macedonian [Jakimovska et al. 2018], German [Meindl 2002], and Norwegian [Heramb et al. 2018]⁠. In Poland, c.3700_3704del has a variable regional distribution. It has been reported in 1.7–6.3% of HBOC patients from northern or north-eastern regions of Poland [Perkowska et al. 2003; Ratajska et al. 2008; Brożek et al. 2008, 2011; Koczkowska et al. 2018]. It is absent or very rare (< 1%) in patients from other provinces [Górski 2006; Brożek et al. 2012; Gaj et al. 2012; Łukomska et al. 2014; Wójcik et al. 2016; Kluz et al. 2018; Kowalik et al. 2018], with the exception of Silesia, where it has been reported in 1.5% of breast cancer patients [Szwiec et al. 2014] (presumably reflecting a short-range gene flow from the neighboring Czechs). The general population frequency of individuals harboring c.3700_3704del has been estimated as 0.1% in the study of 3923 Polish individuals; splitting the study cohort into different regions has confirmed that c.3700_3704del is present only in eastern Pomerania (overlapping with Kashubia) [Brożek et al. 2011]. No or only singular individuals with c.3700_3704del have been reported in other large-scale screens performed in various Polish provinces [Górski 2006; Brożek et al. 2012; Łukomska et al. 2014]. To explain the increased presence of c.3700_3704del in eastern Pomerania in light of its absence/scarcity in the majority of Polish regions, a variety of scenarios, involving historical contacts with Germans, Scandinavians, or Czechs, may be proposed, depending on the dating of the variant’s introduction. Further studies are needed to compare the haplotype background and to estimate age of c.3700_3704del in Kashubs and in other European populations.

## Long-chain 3-hydroxy acyl-CoA dehydrogenase deficiency (LCHADD); *HADHA* c.1528G > C.

Long-chain 3-hydroxy acyl-CoA dehydrogenase deficiency (LCHADD; ORPHA: 5), caused by biallelic pathogenic variants in the *HADHA* gene (OMIM #609016), is a very rare autosomal recessive disease with the worldwide incidence of 1:62,000–1:250,000 [Joost et al. 2011]. If not recognized and treated promptly, it causes high mortality among children [Sims et al. 1995; den Boer et al. 2002; Sykut-Cegielska et al. 2011]. Several studies indicate a more frequent occurrence of LCHADD in Europe, especially around the Baltic Sea basin. In the majority of patients, the disease is caused by the pathogenic *HADHA* variant NM_000182.5:c.1528G > C (p.Glu510Gln), present in compound heterozygosity [IJlst et al. 1996; Tyni and Pihko 1999; den Boer 2000, Sykut-Cegielska et al. 2011; Joost et al. 2012]. The c.1528G > C carrier frequency in the general population has been estimated in many studies: 1:680 in Denmark [den Boer et al. 2000], 1:240 in Finland [Tyni and Pihko 1999], 1:172 in Poland [Piekutowska-Abramczuk et al. 2010], and 1:173 in Estonia [Joost et al. 2012].

The Polish population study [Piekutowska-Abramczuk et al. 2010], on which the c.1528G > C carrier frequency estimate of 1:172 is based, encompassed 6854 Polish neonatal blood samples, including 2976 of Kashubian origin. Calculating the carrier frequency separately for Kashubs (41 carriers detected, 1:73) and the rest of the cohort (18 carriers, 1:217) has revealed significant clustering of c.1528G > C carriers in eastern Pomerania. A similar regional difference in the frequency of c.1528G > C carriers has been reported in a more recent study performed on a large group of adults (1023 Kashubs and 2892 Poles from other provinces, 1:57 versus 1:187, respectively) [Nedoszytko et al. 2017]. The disease incidence predicted from the carrier frequency and the 91% prevalence of c.1528G > C among Polish patients has been estimated at 1:16,900 in eastern Pomerania and 1:118,336 in the general Polish population [Piekutowska-Abramczuk et al. 2010]. This almost seven-fold difference is consistent with the fact that the majority of Polish patients with LCHADD are from the Pomerania district. It is striking when the Kashubs are compared not only to the rest of Poland, but also to other North European regions, including Estonia, which has the highest predicted disease incidence (1:91,700) among the Baltic Sea countries [Joost et al. 2011]. Heralded by the press under the unfortunate name of “Kashubian curse,” the increased frequency of LCHADD triggered a discussion about the stigmatization of the Kashubian minority [Kwaśniewska 2021]. At the same time, however, it has led to launching regional genetic testing, beneficial for potential patients.

The reduction of *HADHA* mutations diversity (c.1528G > C accounting for 91% of the pathogenic *HADHA* variants in Polish patients, the strikingly increased frequency of LCHADD in northern Poland, and the reduced diversity of *HADHA* (c.1528G > C accounting for 91% of the pathogenic variants in Polish patients [Sykut-Cegielska et al. 2011], compared to 87% in other Europeans [IJlst et al. 1996], are consistent with a founder effect in the Kashubian population. To explain when and from which population had the purported founder arrived in this region would require comparing haplotypes associated with c.1528G > C in Poland with those in other Baltic Sea countries.

## Steroid-resistant nephrotic syndrome (SRNS); *NPHS2* c.1032del.

Nephrotic syndrome (NS) is a rare disorder characterized by the increased permeability of the capillary walls of glomeruli, with symptoms such as proteinuria, hypoalbuminemia, hyperlipidemia, and edema. The most common form of treatment in NS is steroid therapy, and although it is effective in most patients, about 10–15% of patients experience the so-called steroid-resistant nephrotic syndrome (SRNS), which is characterized by a lack of response to steroid therapy, ultimately leading to the development of end-stage renal failure in approximately 50% of patients within the next 10 years [Trautmann et al. 2017].⁠ Recent studies have shown that in approximately 30% of patients with SRNS, it is possible to diagnose a monogenic disease as the cause of abnormalities [Trautmann et al. 2020]. Currently, more than sixty genes related to the pathogenesis of SRNS have been described. Biallelic pathogenic variants in the *NPHS2* (OMIM #600995), *NPHS1* (OMIM #256300), or heterozygous pathogenic defects in *WT1* (OMIM #256370) gene are the most common causes [Lipska-Ziętkiewicz 2021].

The *NPHS2* gene variants occur at many geographic locations, e.g., in the Arabs from Israel, in the countries of the Mediterranean Basin, in Spain, or in South America; their distribution and frequencies differ with a population [Bouchireb et al. 2014]. The most common disease-causing *NPHS2* variant in Caucasians, NM_014625.4:c.413G > A (p.Arg138Gln), has been reported in many European populations with frequencies ranging from 1.3 to 9.3% of SRNS chromosomes [Berdeli et al. 2007; Caridi et al. 2009; Megremis et al. 2009; Weber et al. 2004; Ruf et al. 2004; Kerti et al. 2013; Bezdicka et al. 2018]. In the study on 227 Polish SRNS patients, this prevalent European variant has been identified in 2.0% of SRNS chromosomes (4 homozygotes and one carrier), none of them from the Kashubian region [Lipska et al. 2013a]. Another frequent variant, c.686G > A (p.Arg229Gln), considered a risk factor if in *trans* with a pathogenic variant [Caridi et al. 2005; Machuca et al. 2009; Lipska et al. 2013a], is also common in many European populations, with the allele frequency ranging from 2.3 to 5.3% of SRNS chromosomes [Megremis et al. 2009; Ruf et al. 2004, Berdeli et al. 2007; Caridi et al. 2009; Weber et al. 2004; Kerti et al. 2013]. In the study of 141 Polish SRNS children from different regions of the country, the c.686G > A allele has been reported in 5.7% of the chromosomes [Lipska et al. 2013a].

The geographic distribution of another deleterious *NPHS2* variant, c.1032del (p.Phe344LeufsTer4), has a strikingly constrained pattern. The c.1032del allele has been found in 11 of 141 patients from the Kashubian region (2.6% of SRNS chromosomes) [Lipska et al. 2013a]. Besides Kashubs, the c.1032del allele has been reported in one Polish individual from the southern region of the country, in one Caucasian living in Lubeck, Germany [Lipska-Ziętkiewicz, unpublished data], and in two related Caucasian individuals living in the UK [Hinkes et al. 2007; McCarthy et al. 2013]. Considering the fact that Germany and the UK are among the most frequent destinies of the recent migration of Poles, it is tempting to assume that the three non-Polish patients are immigrants of the Kashubian origin. The restricted pattern of the c.1032del occurrence supported by our preliminary analysis of its haplotype background confirms that c.1032del is a Kashubian founder mutation and suggests its very recent origin (manuscript in preparation).

## Perspectives

For most of the diseases presented above, some sort of pre-emptive and/or pre-symptomatic treatment is already available, if the mutation status of a potential patient is known: in FH [Austin et al. 2004; Pang et al. 2020], in LCHADD [Piekutowska-Abramczak et al. 2010], in HBOCS [Rebbeck et al. 2018], in SRNS [Trautmann et al. 2020]).

The increased frequency of certain pathogenic variants in northern Poland is a premise for the implementation of new, region-specific diagnostic procedures and genetic tests, which might lead to more efficient patient-tailored diagnostics and genetic counseling, aiding cascade screening and facilitating informed reproductive decisions. This goal has already been achieved for LCHAD deficiency [Piekutowska-Abramczak et al. 2010], but otherwise, no Kashubian-oriented screening panel is available so far.

The genetic profile of variants characterized by the increased frequency in Kashubs is an example of the diversity of an old ethnic group. Although Kashubs cultivate their language, customs, and traditions, their genetic pool is increasingly blending with the neighboring non-Kashubian people. Therefore, undertaking prompt research into the genetic diversity of this population is very important. Gaining insight into the origin of the still-existing genetic diversity specific to Kashubs offers a possibility to contribute to the knowledge of the history of Slavic peoples’ migrations and demography. The increased frequency of the disease-causing variants in Kashubs has been already documented, but the time of their introduction in the Kashubian population was not examined so far.

The analysis of background haplotypes of the Kashubian variants should be performed to shed light on their age and origin. This information may be essential for developing screening strategies not only in Poland and the neighboring countries, but also in the Kashubian diaspora, who since the second part of the nineteenth century started to settle in Canada, the USA, Brazil, Australia, and New Zealand.

## Footnotes

1 LDLR variants

2 The *BRCA1* variants discussed in this review have been differently referred to in various studies: c.68_69del (185delAG); c.181 T > G (300 T > G; p.Cys61Gly); c.3700_3704del (3819del5); c.4035del (4153delA); c.5266dupC (5382insC).
